# Interdialytic weight gain and educational/cognitive, counseling/behavioral and psychological/affective interventions in patients on chronic hemodialysis: a systematic review and meta-analysis

**DOI:** 10.1007/s40620-022-01450-6

**Published:** 2022-09-16

**Authors:** Maurizio Bossola, Gilda Pepe, Manuela Antocicco, Altea Severino, Enrico Di Stasio

**Affiliations:** 1 Servizio Emodialisi, Policlinico Universitario Fondazione Agostino Gemelli IRCCS, Università Cattolica del Sacro Cuore, Rome, Italy; 2 Dipartimento Scienze Dell’invecchiamento, Neurologiche, Ortopediche e Della Testa-Collo, Rome, Italy; 3 Dipartimento di Scienze biotecnologiche di Base, Cliniche Intensivologiche e Perioperatorie Università Cattolica del Sacro Cuore, Rome, Italy

**Keywords:** Hemodialysis, Interdialytic weight gain, Psychological, Educational/cognitive, Counseling/behavioral, Interventions

## Abstract

**Background:**

This work aimed to shed light on the notorious debate over the role of an educational/cognitive/behavioral or psychological approach in the reduction of interdialytic weight gain (IDWG) in patients on chronic hemodialysis.

**Methods:**

Searches were run from 1975 to January 2022 on Medline, PubMed, Web of Science, and the Cochrane Library. The search terms included “hemodialysis/haemodialysis” AND “adherence” AND (“fluid intake” OR “water intake”) AND (“weight gain” OR “interdialytic weight gain” OR “IDWG”) AND “patient-level interventions. Randomized controlled studies were eligible if they were in English, published in a peer-reviewed journal and regarded adults patients with on chronic hemodialysis for at least 6 months; compared educational/cognitive and/or counseling/behavioral or psychological interventions to no intervention on interdialytic weight gain. Outcome of interest was interdialytic weight gain. The review was registered on the International Prospective Register of Systematic Reviews in Health and Social Care (PROSPERO, ID number CRD42022332401).

**Results:**

Eighteen studies (1759 patients) were included in the analysis. Compared to the untreated group, educational/cognitive and/or counseling/behavioral interventions significantly reduced interdialytic weight gain with a pooled mean difference of − 0.15 kg (95% CI − 0.26, 30–0.05; *P* = 0.004). On the other hand, psychological/affective interventions reduced interdialytic weight gain with a pooled mean difference of − 0.26 kg (95% CI − 0.48, − 0.04; *P* = 0.020).

**Conclusions:**

Educational/cognitive, counseling/behavioral or psychological/affective interventions significantly reduced the interdialytic weight gain in patients on chronic hemodialysis, although such reduction did not appear to be clinically relevant on hard outcomes.

**Graphical abstract:**

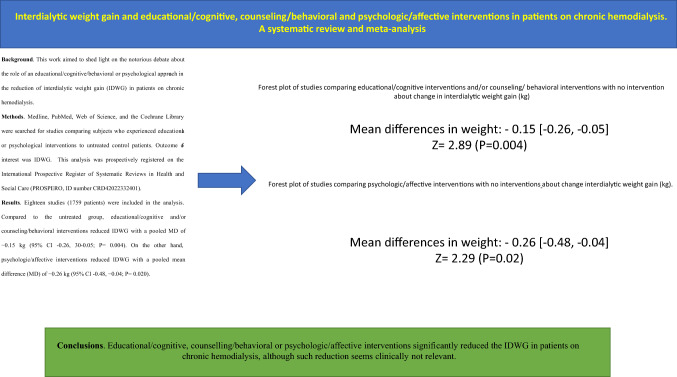

## Introduction

Interdialytic weight gain (IDWG) should be lower than 4.0 to 4.5% of dry weight [1]. Unfortunately, many patients have an IDWG higher than this value [2, 3]. High IDWG is associated with greater risk of all-cause and cardiovascular mortality and increased morbidity, such as ventricular hypertrophy and major adverse cardiac and cerebrovascular events [2, 4–7]. In addition, it leads to supplementary dialysis sessions with consequent reduction of the quality of life and a significant increase in costs.

High IDWG is essentially due to an excessive intake of fluids and/or foods. Non-adherence to both diet and fluid restrictions is very frequent, exceeding 60% of evaluations [8]. Numerous factors have been shown to determine failure to adherence to diet and fluid restrictions [9–18]. Among these, an important role is played by loss of motivation and lack of self-assessment, defined as the inability to correctly define fluid status and salt and fluid intakes [9–18].

In routine clinical practice, improving adherence to restricted fluid intake in patients on chronic hemodialysis is difficult [19, 20]. Among the various strategies that have been attempted to increase adherence to fluid restriction in chronic hemodialysis patients, particular attention has been paid to patient-level interventions that have been categorized according to De Bleser et al. [21] as educational/cognitive (which conveys information or knowledge, individually or in a group setting, and delivers it in a verbal, written, and/or audio-visual form), counseling/behavioral (which targets, shapes and/or reinforces behavior, empowers patients to participate in their care, positively changes a patient’s skill level or normal routine), and psychological/affective (which appeals to the feelings and emotions or social relationships and social supports of the patient).

The present systematic review and meta-analysis aims to evaluate the efficacy of different categories of patient-level interventions in an effort to improve adherence and to limit IDWG in patients on chronic hemodialysis.

According to PICOS criteria, we analyzed: Population = end-stage renal disease patients on chronic hemodialysis; Intervention: educational/cognitive/behavioral treatment; psychological treatment Comparison = no intervention; Outcome = IDWG; Study = systematic review and meta-analysis. The primary outcome of the review is to determine the difference between educational/cognitive or counseling/behavioral or psychological/affective interventions and no interventions in IDGW.

## Methods

This analysis was prospectively registered on the International Prospective Register of Systematic Reviews in Health and Social Care (PROSPERO, ID number CRD42022332401).

### Search strategy

Searches were run from 1975 to January 2022. The following databases were searched for relevant studies: Medline, PubMed, Web of Science, and the Cochrane Library. The search terms and mesh headings included “hemodialysis/haemodialysis” AND “adherence” AND (“fluid intake” OR “water intake”) AND (“weight gain” OR “interdialytic weight gain” OR “IDWG”) AND “patient-level interventions” as the search terms. This review followed the Preferred Reporting Items for Systematic Reviews and Meta-analyses (PRISMA) reporting guideline.

### Eligibility criteria

Studies were eligible for inclusion if they were English language papers published in a peer-reviewed journal and met the following inclusion criteria: (1) primary research studies in adult patients (over 18 years of age), (2) patients with end-stage renal disease on chronic hemodialysis for at least 6 months; (3) compared educational/cognitive and/or counseling/behavioral interventions to no interventions in terms of intradialytic weight gain; (4) compared psychological interventions to no interventions in terms of interdialytic weight gain; (5) included interdialytic weight gain as one of the outcomes of interest. We excluded studies on pediatric patients, pre-dialysis CKD patients, acute kidney injury patients, ESRD patients with other renal replacement therapy modalities such as peritoneal dialysis, and transplantation.

### Data extraction

Two authors (MA and GP) independently reviewed the manuscripts considering the eligibility criteria and quality assessment tools**.** Two authors (M.B. and G.P.) independently reviewed titles and abstracts, and full texts of potential studies were retrieved for further appraisal. In case of disagreement between the two authors, a third author (EDS) was consulted. We also performed a manual search for eligible studies by checking the reference lists of relevant original and review articles. Conference abstracts and literature reviews were excluded. Similarly, studies not comparing standard/low sodium dialysate concentration were excluded. Any discrepancies were resolved by consensus upon discussion with another co-author (EDS). A data extraction table (Table 1) was compiled to record study characteristics and participant characteristics.

**Table 1:** Studies on comparison of educational/cognitive interventions and/or counseling/behavioral interventions to no intervention: effect on IDWG

	Country	Type of study	Number of patients	Intervention	Interventionist	Duration	Outcome/Limits
Cummings et al., 1981 [27]	USA	Randomized, controlled	116	Group A: control Group B: behavioral contract Group C: behavioral contract with a family member or friend Group D: weekly telephone contact	Nurses	3 months	At 3 months, IDWG did not differ among group A (2.6 ± 0.2 kg), group B (2.5 ± 0.2 kg), group C (2.3 ± 0.2), and group D (2.3 ± 0.2 kg) (P = NS)* Demographics of the sample somewhat limit its generalizability
Tanner et al., 1998 [28]	USA	Randomized, controlled	40	Group A: control Group B: patient self-monitoring and behavioral contracting upon adherence	Nurses	6 months	Number of sessions with acceptable IDWG was similar in the two groups
Christensen et al., 2002 [29]	USA	Randomized, controlled	20	Group administered behavioral self-regulation intervention	Nurses	7 weeks	Mean IDWG increased in controls (from 3.12 to 3.3 kg) and decreased in treated patients (from 3.12 to 2.9 kg) (P = 0.06) Short study duration; small sample
Molaison and Yadrick, 2003 [30]	USA	Comparison of patients at two centers (one as treatment and one as control)	316	Group education sessions based on transtheoretical model (states of change)	Multiple	3 months	Mean IDWG increased both in controls (from 3.44 ± 1.27 kg to 3.57 ± 1.21 kg) and in treated patients (from 3.24 ± 1.19 to 3.41 ± 1.14 kg) (P = NS)*
Tsay, 2003 [31]	Taiwan	Randomized, controlled	62	Self-efficacy training	Nurses	3 weeks	Mean IDWG decreased in controls from 2.6 ± 0.7 to 2.5 ± 0.9 kg and in treated patients from 3.3 ± 0.7 to 2.5 ± 0.7 kg (P = 0.006)* Short study duration
Sharp et al., 2005 [32]	UK	Randomized, controlled	56	Group-based cognitive behavioral intervention	Psychologist	1 month	Small but significant decrease in IDWG in the treatment group: from 3.56 ± 0.91 at baseline to 2.96 ± 1.09 kg at 4 weeks*
Kauric-Klein et al., 2012 [33]	USA	Randomized, controlled	118	Supportive nursing intervention incorporating monitoring, goal setting, and reinforcement	Nurses	4 months	No differences in IDWG
Cho et al., 2013 [34]	Korea	Randomized, controlled	43	Health contract intervention based on the goal attainment theory	Nurses	1 month	IDWG lower in the experimental group (P = 0.017) Short study duration
Welch et al., 2013 [35]	USA	Randomized, controlled	44	Electronic self-monitoring intervention based on social cognitive theory	Nurses	6 weeks	No differences in IDWG Short study duration
Cukor et al., 2014 [36]	USA	Randomized, crossover	59	Individual cognitive behavioral therapy	Psychologist	3 months	Percentage of change in weight per day in controls was 3.6 at baseline and 2.5 at 3 months and in the treatment group it was 4.0 at baseline and 3.6 at 3 months (P = 0.002)
Griva et al., 2018 [37]	UK	Randomized, controlled	235	Interactive and targeted self-management training program	Multiple	9 months	IDWG significantly (P < 0.001) lower in experimental group
Baser et al., 2018 [38]	Turkey	Randomized controlled	78	The participants in the intervention group were trained through four education sessions over 4 months, and the measurement tools were administered to them	Nurses	4 months	Mean IDWG decreased in controls from 2.3 ± 1.4 to 2.2 ± 1.9 kg (P = 0.772) and in treated patients from 3.2 ± 1.7 to 1.8 ± 1.1 kg (P = 0.0001)

Data are expressed as * mean ± SD

### Statistical analysis

Statistical analysis was performed using the Statistical Package for Social Science (SPSS 22.0; SPSS Inc, Chicago, IL, United States) and Microsoft Excel. The primary outcome of the review is to determine the difference between educational/cognitive or counseling/behavioral or psychological/affective interventions and no interventions in the IDGW (mean difference); each meta-analysis (Forest plot) was built using studies enrolling one of the two therapeutic approaches compared to untreated subjects, and the mean difference (random effect), weight of the single study and heterogeneity parameters (Tau, Chi^2^, p, I^2^ and Z and p for overall effect) are reported. Statistical heterogeneity among studies was quantified with Higgins *I*^2^ statistic. Publication bias was assessed graphically using funnel plots.

## Results

### Search results

**Fig. 1:**
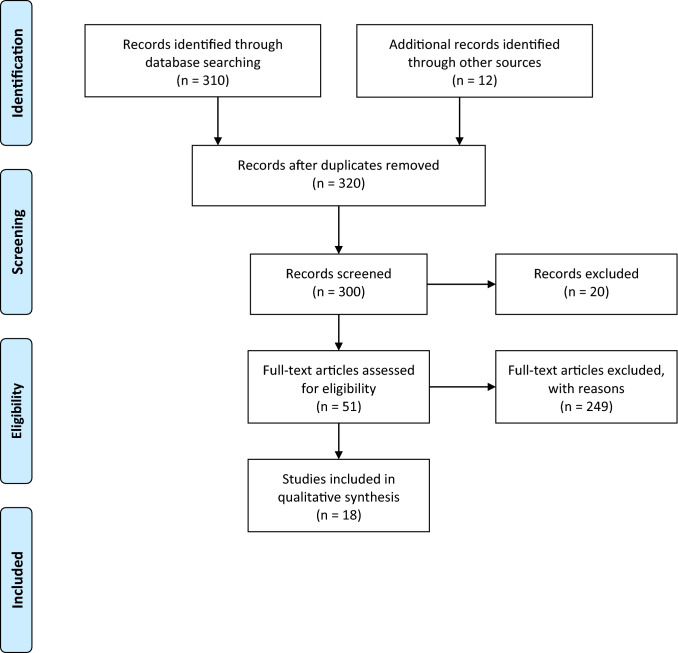
Preferred Reporting Items for Systematic Reviews and Meta-Analyses (PRISMA) flowchart of our analysis

A total of 320 publications were identified via electronic databases. After screening titles, abstracts, and full texts, 18 studies meeting the inclusion criteria were included for analysis. The PRISMA flowchart is shown in Fig. 1. All studies were randomized and controlled. We divided the analysis into two sections: (1) Comparison of educational/cognitive/counseling/behavioral interventions versus no intervention, in terms of IDWG (12 studies); (2) Comparison of psychological/affective interventions versus no intervention in terms of IDWG (6 studies).

### Study characteristics

#### Educational/cognitive interventions and/or counseling/behavioral interventions

Overall, 1187 patients were included. The number of patients in each individual study ranged from 20 to 316. The length of the studies ranged from 3 weeks to 9 months and a description is presented in Table 1 [27–38]. In the majority of studies, educational/cognitive interventions were performed in combination with counseling/behavioral interventions [30–33, 35, 36, 38], whereas in four studies, counseling/behavioral interventions were delivered [27–29, 34] as the sole strategy. Moreover, educational/cognitive and counseling/behavioral interventions were generally performed through an individual format, except in two studies where a group format was used [32, 37].

#### Psychological/affective interventions

Overall, 572 patients were included. The number of patients in each individual study ranged from 67 to 119. The length of studies ranged from 5 weeks to 12 months and a description is presented in Table 2 [39–44]. In most studies, psychological/affective interventions were performed through an individual format, except in one study where a group format was used [42].

**Table 2:** Studies on comparison of psychological/affective interventions to no intervention: effect on IDWG

	Country	Type of study	Number of patients	Intervention	Interventionist	Duration	Outcome/Limits
Hou et al., 2010 [39]	China	Randomized, controlled	92	Rational emotive therapy based on ABC theory	Psychologist	3 months	Significant decrease in IDWG at 3 months in the treatment group Relatively small sample size
Pasyar et al., 2015 [40]	Iran	Randomized, controlled	86	Relaxation technique	Professional relaxation therapist	2 months	Small but significant decrease in IDWG at 4 months in the treatment group Exclusion of patients with unstable hypertension, angina, arrhythmia, congestive heart failure, acute cerebrovascular accident, hepatic failure Relatively small sample size
Bellomo et al., 2015 [41]	Italy	Randomized, controlled	117	Group sessions held once a week	Psychologist	5 weeks	IDWG decreased from baseline to 6 months: in controls, from 1.31 ± 0.33 to 1.32 ± 0.32 kg (*P* = 0.57); in treatment group, from 1.33 ± 0.33 to 1.2 ± 0.28 kg (*P* < 0.001) * Short study duration
Howren et al., 2016 [42]	USA	Randomized, controlled	119	Behavioral self-regulation intervention	Psychologist	7 weeks	No differences between groups in mean IDWG Short study duration Information regarding patient expectations or motivation were not collected or reported here
Wileman et al., 2016 [43]	UK	Randomized, controlled	91	Self-affirmation theory to reduce resistance to health-risk information	Psychologist	12 months	Small but significant decrease in IDWG at 12 months in the treatment group Relatively small sample size Residual kidney function not assessed
Valsaray et al., 2021 [44]	India	Randomized, controlled	67	Cognitive behavior therapy	Nurse	6 months	IDWG changed from baseline to 6 months: in controls, from 4.3 ± 0.7 to 4.6 ± 0.4 kg (P = NS); in treatment group, from 4.4 ± 0.9 to 3.2 ± 0.6 kg (P = 0.001) Relatively small sample size

Data are expressed as * mean ± SD

### Efficacy of interventions on IDWG

#### Educational/cognitive interventions and/or counseling/behavioral interventions

Nine of the 12 studies comparing educational/cognitive interventions and/or counseling/behavioral interventions to no intervention were included in the meta-analysis.

**Fig. 2:**
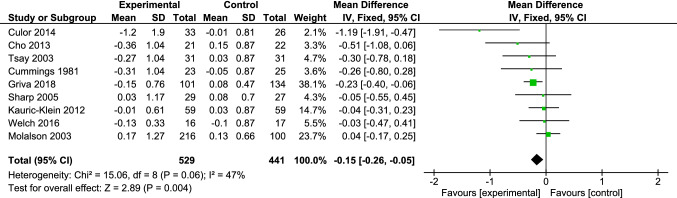
Forest plot of studies comparing educational/cognitive interventions and/or counseling/ behavioral interventions to no intervention with regard to change in interdialytic weight gain (kg)

As shown in Fig. 2, compared to no intervention, educational/cognitive/counseling/behavioral interventions reduced IDWG, with a pooled mean difference (MD) of − 0.15 kg (95% CI − 0.26, − 0.05; P = 0.004). As no significant heterogeneity was observed (Chi^2^ = 15.06; *I*^2^ = 47%; *P* = 0.06), the pooled analysis was performed using a fixed-effect model.

Three studies were not included in the meta-analysis because IDWG was not expressed as mean (SD) difference between pre- and post-treatment values [10, 28, 38]. In the study by Tanner et al., the number of sessions with acceptable IDWG was similar in the two groups [28]. In the study by Christensen et al., mean IDWG increased in controls (from 3.12 to 3.3 kg) and decreased in treated patients (from 3.12 to 2.9 kg), but the difference was not statistically significant (*P* = 0.06) [29]. In the study by Baser et al., mean IDWG decreased in controls from 2.3 ± 1.4 to 2.2 ± 1.9 kg (*P* = 0.772) and in treated patients from 3.2 ± 1.7 to 1.8 ± 1.1 kg (*P* = 0.0001) [38].

#### Psychological/affective interventions

Four of the 6 studies comparing psychological/affective interventions to no intervention were included in the meta-analysis.

**Fig. 3:**
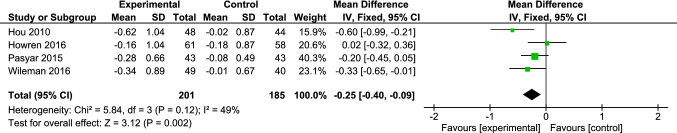
Forest plot of studies comparing psychological/affective interventions to no interventions with regard to change in interdialytic weight gain (kg)

As shown in Fig. 3, compared to no intervention, psychological/affective interventions reduced IDWG with a pooled MD of − 0.26 kg (95% CI − 0.48, − 0.04; *P* = 0.002). As no significant heterogeneity was observed (Chi^2^ = 5.84; *I*^2^ = 49%; *P* = 0.12), the pooled analysis was performed using a fixed-effect model.

Two studies were not included in the meta-analysis because IDWG was not expressed as mean (SD) difference between pre- and post-treatment values [21, 44]. In the study by Bellomo et al., IDWG changed from baseline to 6 months; in controls it ranged from 1.31 ± 0.33 to 1.32 ± 0.32 kg (P = 0.57) and in the treatment group it decreased from 1.33 ± 0.33 to 1.2 ± 0.28 kg (*P* < 0.001) [41]. In the study by Valsaray et al., the IDWG changed from baseline to 6 months; in controls it went from 4.3 ± 0.7 to 4.6 ± 0.4 kg (*P* = 0.856) and in the treatment group it dropped from 4.4 ± 0.9 to 3.2 ± 0.6 kg (*P* = 0.001) [44].

### Publication bias

**Fig. 4:**
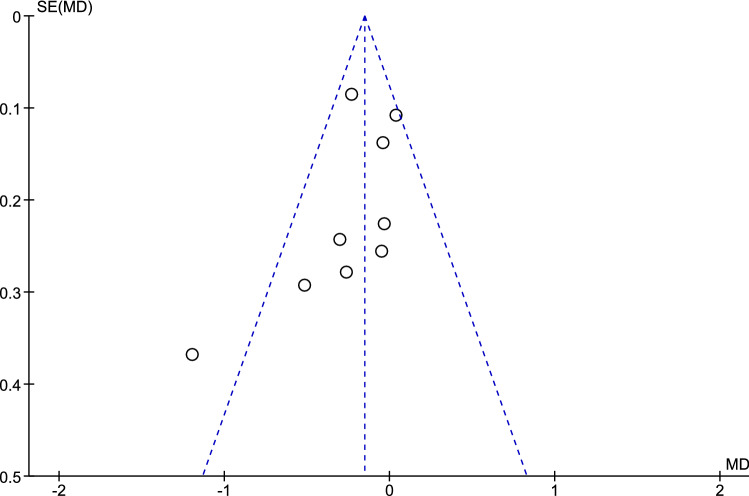
Funnel plot of studies comparing educational/cognitive interventions and/or counseling/behavioral interventions to no intervention

**Fig. 5:**
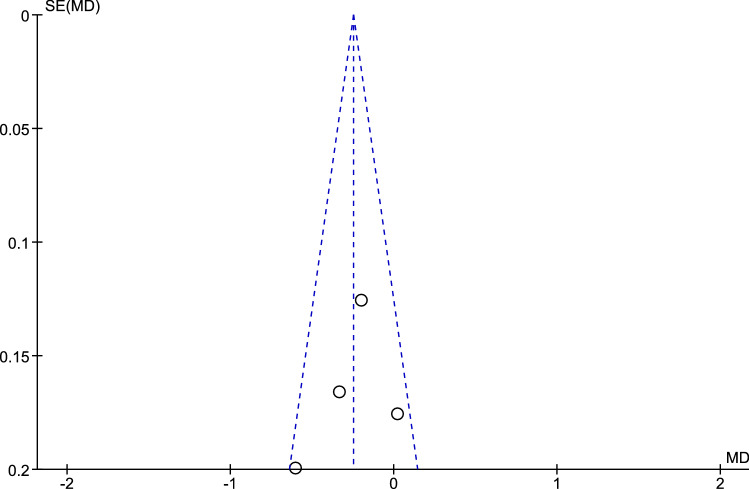
Funnel plot of studies comparing psychological/affective interventions to no interventions

Funnel plots were generated to assess publication bias in the included studies. No obvious asymmetry, which indicated no clear evidence of publication bias, was observed either in the studies comparing educational/cognitive interventions and/or counseling/behavioral interventions to no intervention (Fig. 4) or in the studies comparing psychological/affective interventions to no interventions (Fig. 5).

## Discussion

The meta-analysis of 9 and 4 randomized studies shows that educational/cognitive interventions and/or counseling/behavioral interventions or psychological/affective interventions are both effective in significantly reducing IDWG in patients on chronic hemodialysis; the pooled mean difference of IDWG was reduced by − 0.15 and − 0.26 kg, respectively. In addition, the review of studies not included in the meta-analysis reveals conflicting results in both treatment approaches.

The results of the present meta-analysis are in agreement with the recent work by Murali et al. in which IDWG was significantly reduced as the effect of patient-level or health system-related interventions, with a pooled IDWG reduction of − 0.20 [− 0.32 to − 0.081]; it is important to note that in Murali’s study, patient-level interventions were considered as an individual entity, without distinguishing among educational/cognitive, counseling/behavioral, and psychological/affective interventions [46].

However, it could be questioned whether such differences in weight actually reflect a clinically relevant effect. Interestingly, other interventions have led to better results in terms of reduction of IDWG. In fact, a recent systematic review and meta-analysis showed that the use of a low dialysate sodium concentration significantly reduced the IDWG in prevalent patients on chronic hemodialysis, with a pooled mean difference of − 0.42 kg (*P* < 0.00001) [47]. In addition, the large study by Marshall et al. showed that the use of low dialysate sodium concentration led to a sustained decrease in IDWG (− 0.56 kg and − 0.61% of pre-dialysis body weight) accompanied by an early decrease in extracellular fluid volume [48]. Interestingly, during a 10-year period, between 2004 and 2014, in the Dialysis Outcomes and Practice Patterns Study (DOPPS) [2], both absolute and relative IDWG decline were − 0.29 kg and − 0.5% of post-HD weight in the United States, − 0.25 kg and − 0.8% of post-HD weight in Canada, and − 0.22 kg and − 0.5% of post-HD weight in Europe, respectively. The DOPPS study also demonstrated that the dialysate sodium concentration accounted for 0.13 greater relative IDWG per 1-mEq/L greater dialysate sodium concentration, suggesting that it played a relatively important role in explaining the decline in IDWG [48].

Low dialysate sodium concentration results in greater diffusive sodium removal during dialysis with consequent lower total body sodium content by the end of treatment, which might therefore lessen thirst and water intake in the interdialytic period. This in turn might reduce extracellular fluid overload, hypertension, and ultimately, left ventricular hypertrophy and CV death. In daily clinical practice, dialysate sodium concentration may be fixed (low or high) or variable (individualized). High dialysate sodium concentrations provide hemodynamic benefits and prevent hypotensive episodes, but reduce the loss of sodium and consequently, thirst is stimulated and the weight gain increases. However, it should be kept in mind that the use of a low dialysate sodium concentration may be in some cases associated with adverse events such as muscle cramps and intra-dialytic or post-dialytic hypotension, which are the result of intradialytic hemodynamic instability [2]. Thus, the final decision to adopt a low dialysate sodium concentration depends on the clinical evaluation and assessment of costs and benefits.

Patient-level interventions are expensive, time consuming and require the not always achievable cooperation of patients [20, 21, 27, 28]. Educational/cognitive, counseling/behavioral or psychological/affective interventions often need multiple sessions over time [20, 21]. Educational/cognitive interventions require videos, posters, and presentations to improve patient education. In addition, the educational content of such interventions is complex and includes information about the nature of the renal disease and the consequences of renal insufficiency, the physiology of thirst, and the consequences of high salt and excessive fluid intake [20, 21]. Finally, the acquisition of knowledge is not necessarily associated with behavioral changes. Counseling/behavioral interventions require continuous reinforcements, directly or via phone call, regular feed-back and contacts at home [20, 21, 29–32]. Often, once adherence has been achieved, there is a risk of recurrence of non-adherence [20, 21, 27–32]. Psychological/affective interventions are based on the intervention of a psychologist. Unfortunately, this action is not available in many hemodialysis units.

Finally, it is unclear whether patient-level interventions need to be continued at length in order to have a clinically meaningful effect [27–42]. Indeed, the duration of the studies included in the present review ranged between 3 weeks and 12 months. In addition, it remains unknown if the reduction of IDWG persists after the interruption of the patient-level interventions. In fact, none of the studies reported the long-term impact of patient-level interventions after their cessation. This is a key point that needs to be clarified by adequate, randomized, controlled studies in the near future**.**

In light of these considerations, and of the clinically irrelevant IDWG reduction, the role of patient-level interventions aimed at reducing weight gain in patients on chronic hemodialysis should be questioned. The issue remains whether, it is worth continuing these interventions in routine clinical practice in an attempt to limit IDWG in patients on chronic hemodialysis. Overall, it seems that further, large, randomized controlled studies are warranted to reach a definitive result.

However, the present review highlights some interesting observations. It focuses on fluid adherence, a fundamental aspect of patients on chronic hemodialysis, as measured by IDWG, and considers only patient-level interventions categorized as educational/cognitive/counseling/behavioral interventions and psychological/affective interventions.

The present review has some limitations. First, the sample size of many of the included trials was small. Second, the length of the studies was extremely varied and short, ranging from one month to twelve months. Third, the residual urine volume was not reported in all studies. Finally, data collected from the literature could be affected by some biases (different operators, substantially non-numerable parameters, other national health systems).

In conclusion, the present meta-analysis shows that educational/cognitive interventions and/or counseling/behavioral interventions or psychological/affective interventions are effective in reducing IDWG in patients on chronic hemodialysis**.** However, the absolute IDWG reduction associated with these interventions seems to be of limited relevance in the clinical setting. Thus, more studies are warranted to improve the efficacy of educational/cognitive, counseling/behavioral or psychological/affective interventions in reducing IDWG.
